# Water Footprint of Food Consumption by Chinese Residents

**DOI:** 10.3390/ijerph16203979

**Published:** 2019-10-18

**Authors:** Yu Zhang, Qing Tian, Huan Hu, Miao Yu

**Affiliations:** 1 School of Geography and Tourism, Qufu Normal University, Rizhao 276826, China; zhangyu_0730@163.com; 2 College of Economic and Management, Tongren Polytechnic College, Tongren 554300, China; bingquan1987@163.com (Q.T.); yumiao1918@126.com (M.Y.); 3 School of Economics and Management, Hangzhou Normal University, Hangzhou 311121, China

**Keywords:** water footprint, virtual water, water shortage, food consumption, permanent residents

## Abstract

Water shortages are a worldwide problem. Virtual water and the water footprint link water resources, human beings and agricultural products, and are effective tools to alleviate water-resources stress. The production of agricultural products consumes a large amount of water, and food is the most basic consumer good for human survival, so it is very necessary to study the water footprint of residents’ food consumption, which is also the weak point of current research on virtual water and the water footprint. This paper aimed to conduct a comprehensive analysis on the water footprint of food consumption in China from the perspectives of urban and rural residents, per capita water footprint, water footprint structure and food consumption structure. The results revealed that the average water footprint of residents’ food consumption was 605.12 billion m3/year, basically showing an upward trend. Guangdong residents had the highest water footprint for food consumption due to the highest population and higher consumption of water-intensive foodstuffs such as grain and meat in their diet. The water footprint of Xizang residents’ food consumption was the lowest followed by Ningxia and Qinghai due to having the least population. The water footprint of food consumption consumed by urban residents was on the rise while that consumed by rural residents was on the decline in China, which was consistent with the changing trend of population. On the whole, the rural population consumed more virtual water embedded in food than the urban population. From the water footprint structure point, the contribution rate of the green water footprint is the largest, reaching 69.36%. The second is the gray water footprint and then the blue water footprint, accounting for 18.71% and 11.93%, respectively. From the perspective of the food consumption structure, grain and pig, beef and mutton consumption contributed significantly to the total water footprint of residents’ food consumption, contributing 37.5% and 22.56%, respectively. The study is helpful for water management and water allocation in rural and urban areas, improving agricultural technology to reduce the gray water footprint and optimizing food consumption structure, such as reducing the consumption of grain and meat.

## 1. Introduction

Freshwater is crucial for human well-being and sustainable socio-economic development. With the continuous development of the economy, urbanization, industrialization, population growth and changing consumption pattern, water scarcity has become increasingly prominent [1]. The water resource crisis and energy crisis are two major crises facing mankind in the 21st century [2,3,4]. *The Global Risks Report 2017* assesses the impact of global risks, with the water crisis ranking third [5]. Freshwater withdrawals have increased globally by about 1% per year since the 1980s [6]. Global water demand (in terms of water withdrawals) is projected to increase by some 55% by 2050, and more than 40% of the global population is projected to be living in areas of severe water stress through 2050 [7]. The World Bank predicts that urban water supplies could fall by as much as two-thirds as a result of climate change and competition between energy generation and agriculture [8]. 

A core solution to this problem is to strengthen the management of water resources and improve water-use efficiency. However, traditional water resources management only refers to the direct water use of agriculture, industry and residential life, which cannot reflect the real water consumption. Virtual water and the water footprint are considered as effective tools to alleviate the water crisis [9]. In 1993, Tony Allan innovatively proposed the concept of “virtual water” to describe the amount of water consumed in the production of products or services when explaining water problems in the Middle East. Virtual water is different from the physical water resources in the public concept. The characteristics of virtual water lie in “virtual” and “invisible”, also known as embodied water, embedded water and invisible water [10,11]. The concept of the water footprint is based on the concept of virtual water, which was proposed by Hoekstra in 2002. It refers to the amount of water resources required to produce products and services consumed by a certain population under certain material living standards. It represents the real amount of water resources, including physical water and virtual water [12,13]. Since the concept of the water footprint was put forward, it has become a hot topic in current water resources research, and has drawn much attention in the field of hydrology and water resources. 

Water footprint research mainly focuses on the measurement and evaluation of the water footprint at different scales, such as the global scale, national scale and watershed scale. On a global scale, Hoekstra and Hung first studied the water footprint of each country on a global scale in 2002 [12]. Subsequently, Hoekstra and Chapagain et al. conducted a second, more comprehensive study and reported it in a number of subsequent publications [14,15,16,17,18]. The third global assessment of the country’s water footprint was conducted by Mekonnen and Hoekstra in 2011. It is the NO.50 report published by United Nations Educational, Scientific and Cultural Organization (UNESCO)–IHE Institute for Water Education, which improves on previous assessments in several ways, taking into account green water, blue water and gray water in a more comprehensive and detailed way [19]. In addition to global water footprint studies, there have been several country-specific water footprint studies in the past few years. For example, Belgium [20], Germany [21], India [22], Indonesia [23], Morocco [24], The Netherlands [24], Iran [25], Spain [26], Tunisia [27], New Zealand [28], Uzbekistan [29], Turkey [30] and China [31,32,33,34]. However, the scope, assumptions and sources of data of these country studies vary greatly and cannot be used for country comparisons. Watershed water footprint research is also a hotspot. Dumont analyzed the green and blue water footprint of the Guadalquivir basin and its relationship with environmental water consumption, focusing on the analysis of groundwater footprint and its impact on current and future surface water depletion [35]. Francisco Pellicer-Martínez et al. proposed a basin water footprint calculation method and took Segura River Basin in southeast Spain as an example to conduct an empirical study [36]. Zeng et al. quantitatively studied the water footprint of China’s Heihe River basin and found that the annual average water footprint of the heihe river basin from 2004 to 2006 was 1768 million m^3^, and the agricultural production was the largest contributor to the water footprint [37].

In terms of products, the current research on water footprint is mainly about the measurement of the water footprint of agricultural products or the research on water footprint based on agricultural products. Reports 47 and 48 of the United Nations Institute for Water Education, for example, systematically study the water footprints of agricultural products in countries around the world [38,39]. There are also studies on the water footprint of specific agricultural products, for example, cotton [40], meat and milk [41], rice [42,43], maize [44], tea and coffee [45], potato [46,47], Pigs, beef and mutton [48,49], sugar cane and cassava [50]. At present, there are also a lot of studies on the water footprint of the secondary and tertiary industries, for example, transport fuels [21], biofuels [51,52,53,54], electricity [55,56], wine making [57,58], soy milk and soy bueger [59], soap bar [60], gaming industry [61], food company [62], tourism [63,64,65,66,67].

The dietary water footprint of residents is also one of the research hotspots. Hou et al. conducted a quantitative study on the dietary water footprint of urban and rural residents in three northeastern provinces from 2000 to 2013, and found that the per capita water footprint of urban residents was on the rise while that of rural residents was on the decline [68]. After studying the water footprint consumed by Beijing residents, Wu et al. found that the virtual water consumed per capita is five times more than the real water, and the food supply transferred from other provinces in China can reduce the virtual water consumption [69]. With the improvement of people’s income level, the dietary structure is constantly changing, leading to the change of water footprint. Qin et al. conducted a quantitative study on the dietary water footprint of urban residents with different income levels in Jilin city, and the results showed that the per capita dietary water footprint of the lowest income was the lowest, and the per capita dietary water footprint of the highest income was the highest [70]. However, the exsiting literature on the water footprint of food consumption mainly concentrated on several provinces, and the comprehensive analysis of each province needs to be enriched. 

At present, China’s water resources have different degrees of bottlenecks in terms of type, use, time and space, and the coordination of groundwater, surface water, intermediate water and new water, and the competition of water for agriculture, industry and tertiary industry. On the whole, the contradiction between supply and demand of water resources is very prominent, and water pollution is serious. Agriculture is a large consumer of water resources in China. From 2003 to 2016, the average annual agricultural water use was 371.3 billion m^3^, accounting for about 63% of the total water use [71]. Water footprint links water resources, agricultural products and human beings. Moreover, China has a large population and food consumption is the largest grain use in China. Therefore, the analysis of the water footprint of residents’ food consumption plays an important role in reducing the overall water footprint. It is also an important reference to adjust the food consumption structure of residents. 

The aim of this paper is to conduct a diachronic analysis on the water footprint of residents’ food consumption in China from 2001 to 2016 and to analyze the water footprint of food consumption in each province, from the perspective of rural and urban residents, per capita water footprint, water footprint structure and food consumption structure. The paper takes crop and livestock products into consideration, considering 7 crop products and 5 animal products. Following the introduction, Section 2 explains the methodology used and introduces the data sources. The results and discussion are analyzed in Section 3, and then the conclusions are presented in Section 4.

## 2. Methods and Data

### 2.1. Methods

#### 2.1.1. Water Footprint Model of Residents’ Food Consumption

The water footprint of residents’ food consumption has been calculated by multiplying the per food quantity consumed by the residents by their associated water footprint per ton of product.

$${\mathrm{WF}}_{food}=\overset{n}{\underset{i=1}{\sum }}(Q_{i}*F_{i})$$

${\mathrm{WF}}_{food}$ denotes the water footprint of residents’ food consumption in China; $Q_{i}$ denotes the quantity of food *i* the residents consumed (ton); $F_{i}$ denotes the water footprint per unit of food *i* (m^3^/ton).

#### 2.1.2. Food Consumed

According to the dietary structure and the availability of food consumption data of residents, 12 kinds of food such as rice, wheat, corn, soybean, potato, vegetable, edible vegetable oil, pork, beef, mutton, poultry and eggs were selected as research objects.

#### 2.1.3. Water Footprint Structure

According to the sources of water, the water footprint can be divided into green water footprint, blue water footprint and gray water footprint. Green water footprint refers to the rainwater consumed in the production of a good, blue water footprint refers to the surface and groundwater consumed (evaporated), and gray water footprint denotes the water pollution, the volume of freshwater that is required to assimilate the load of pollutants based on existing ambient water-quality standards. The gray water footprint of crop production, which is an indicator of the volume of freshwater pollution, is calculated by quantifying the volume of water needed to assimilate the nutrients that reach ground or surface water [38].

### 2.2. Data

#### 2.2.1. Water Footprint Per Unit of Food Consumed

Different agricultural products use different amounts of water. The same agricultural products will also consume different amounts of water resources due to regional differences in climatic conditions, production technology, and soil characteristics. At present, the most comprehensive and detailed research on the water footprint of agricultural products is the reports Nos. 47 and 48 published by Mekonnen, M.M. and Hoekstra, A.Y., which involves the water footprint of different agricultural products in various countries and administrative regions around the world. Due to the unavailability of data, we cannot calculate all the 12 categories of agricultural products in all provincial-level administrative regions of China, so we directly use the relevant data in report 47 and 48 [38,39]. It should be noted, however, that the reported results are based on data from 1996–2005. Since 2005, there have been many studies on the measurement of water footprint of agricultural products, but due to different parameter settings or methods, it is not easy to do comparative studies. Although the report has some limitations, it can also provide some references for policy makers or managers or researchers because it is equal measurement.

The 12 categories of agricultural products include 7 categories of crop products and 5 categories of animal products (Table 1). Water footprint of rice, wheat, corn, soybean and potato can be obtained directly from report No. 47. The water footprint of vegetables was an average of 20 kinds of vegetables. The water footprint of edible vegetable oil was an average of 11 kinds of vegetable oil. The water footprint of animal products includes three aspects: animal feed water, animal drinking water and service water. Service water includes water for cleaning and water necessary to maintain the environment [39]. Assuming that the drinking water and service water of animals are the same in each province, the difference of water footprint of animal products in each province is mainly reflected in the difference of grain water footprint in the feed. The main sources of grain for feed are corn flour, bran, and bean cakes. On the premise that the national average water footprint of all kinds of animal products is known, the water footprint of corn flour, bran and soybean cake and the proportion of their national average water footprint are taken as the proportion of the water footprint of animal products in each province and the national average, and then the water footprint of animal products in each province can be calculated. In addition, the water footprint per unit of the bran is based on the data of flour, which is also the product of wheat grinding and they have the same water footprint.

#### 2.2.2. Residents’ Food Consumption

In China’s statistical yearbook and provincial statistical yearbook, there are detailed records of residents’ consumption, such as the annual consumption of different food for each person in urban and rural areas. In this study, data from 2001 to 2016 were used for a diachronic analysis, and the missing data were calculated by using the existing data. The per capita food consumption and water footprint of urban and rural residents in each province multiplied by the population of urban and rural residents is the annual food consumption and water footprint of the urban and rural population in each province. We did not calculate the imported agricultural products, because the yerbooks did not distinguish where the food came from. Moreover, the imported agricultural products were mainly used as feed grain and industrial grain. 

## 3. Results and Discussion

### 3.1. Comprehensive Analysis 

**Figure 1:**
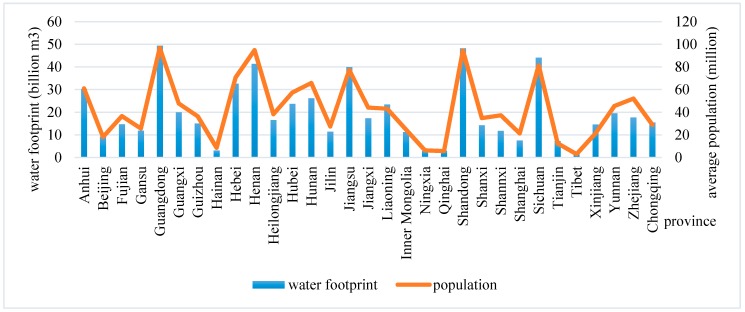
The average water footprint of residents’ food consumption and population.

From 2001 to 2016, the total water footprint of residents’ food consumption was 9681.86 billion m^3^ and the average was 605.12 billion m^3^/year, basically showing an upward trend. Guangdong residents have the highest water footprint for food consumption, at 49.37 billion m^3^/year. Then the following were Shandong province and Sichuan province, the average annual water footprint of residents’ food consumption was 48.24 billion m^3^/year and 44.07 billion m^3^/year respectively. These three areas had highest population and consumed more water-intensive products, such as grain and meat. The water footprint of Xizang residents’ food consumption was the lowest, only 1.70 billion m^3^/year, and then followed by Ningxia and Qinghai. These three areas also had the least population, with 2.95 million, 6.21 millon and 5.58 million respectively. The average water footprint of residents’ food consumption was related to the population (Figure 1). 

The water footprint of residents’ food consumption is the product of the number of residents’ food consumption and the food water footprint per unit. Therefore, the water footprint per unit of food consumption is very important for reducing the overall food consumption water footprint of residents. The water problems of agricultural production must be put forward. Globally, agriculture is a major water consumer. According to statistics, the annual global virtual water trade reached 986.7 billion m^3^, equivalent to the flow of 20 Niles. Two-thirds of that is found in a variety of crops; A quarter is found in meat and dairy products; only one tenth exists in industrial products [72]. Moreover, the distribution of water resources between agriculture and industry and between rural and urban areas is uneven. When water resources are in short supply, demand for food production always follows. Agricultural and rural water use is the largest and most needed and in fact it is the least managed part of China’s water use. The water shortage in agriculture and rural areas is actually much more severe than residents perceive [73].

In the production process of agricultural products, the difference of water footprint is caused by the difference of natural conditions such as climate in different provinces and the level of agricultural production technology. To reduce the water footprint of agricultural production and improve agricultural production levels, water-saving irrigation is the most important. At present, China’s agricultural production water diversion equipment and irrigation technology are relatively backward compared with developed countries, and most of them adopt flood irrigation. The proportion of farmers using water-saving irrigation in land areas is relatively low, and the agricultural water-use efficiency is low. China should actively learn from the advanced technologies of foreign developed agriculture, improve the level and efficiency of irrigation, and save water. In addition, corresponding water management organizations should be established to improve farmers’ awareness and desire to independently manage water resources [73]. 

### 3.2. Water Footprint of Urban and Rural Residents’ Food Consumption 

Due to different living standards and habits, there are differences in food consumption structure between urban and rural residents. In addition, due to the differences in planting structure, natural conditions and production levels in different areas, the water footprint of each unit of agricultural products is different. This has led to differences in the amount of water resources consumed by urban and rural residents in different provinces, which should be analyzed differently.

**Figure 2:**
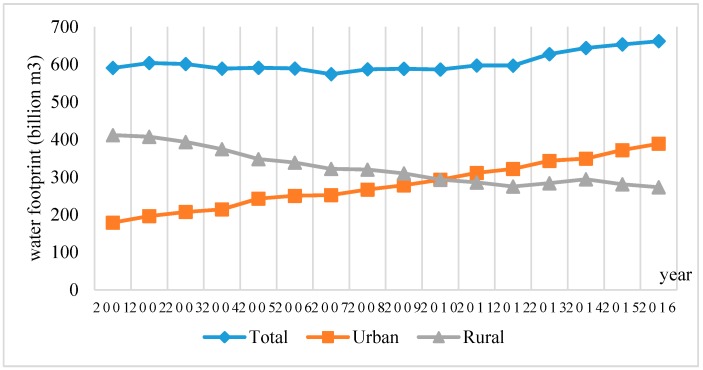
The trend of the water footprint of food consumption.

After calculation, the total amount of water footprint consumed by urban residents in China was on the rise from 2001 to 2016 (Figure 2). This is consistent with the changing trend of urban residents. The population of urban areas was increased from 0.44 billion in 2001 to 0.80 billion in 2016, and the proportion has decreased from 35% to 58% of the total population correspondingly. The total amount of water footprint consumed by urban residents in all provinces was 4467.61 billion m^3^, with an annual average of 279.23 billion m^3^. Urban residents in Guangdong province consumed the highest water footprint with an annual average of 27.57 billion m^3^, followed by Shandong province at 21.57 billion m^3^ and Jiangsu province at 20.69 billion m^3^. The water footprint of urban residents’ food consumption in Tibet was the lowest at 0.42 billion m^3^ per year, followed by Qinghai province at 1.11 billion m^3^ and Ningxia at 1.21 billion m^3^.

From 2001 to 2016, the water footprint of food consumption of rural residents in China has been on the decline. This is consistent with the trend of rural residents. The population of rural areas was declined from 0.81 billion in 2001 to 0.58 billion in 2016, and the proportion has decreased from 65% to 42% of the total population correspondingly. The total amount of virtual water consumed by rural residents in all provinces is 5214.25 billion m^3^, with an annual average of 325.89 billion m^3^. Among all provinces, the rural population in Sichuan province consumes the most virtual water with an annual average of 29.59 billion m^3^, followed by Shandong province and Guangdong province with an annual average of 26.68 billion m^3^ and 21.80 billion m^3^, respectively. The rural population in Shanghai consumed the least amount of virtual water with an annual average of 1.26 billion m^3^, followed by Tibet and Beijing with an annual average of 1.28 billion m^3^ and 1.42 billion m^3^, respectively.

The rural population consumes more virtual water embedded in food than the urban population. This is related to the food consumption structure and the quantity of food consumed by urban and rural residents. The grain consumption of urban residents is significantly lower than that of rural residents. For example, in 2011, the average grain consumption of urban residents in China was 105.89 kg, while that of rural residents was 126.96 kg [74]. 

### 3.3. Per Capita Water Footprint of Residents’ Food Consumption 

**Figure 3:**
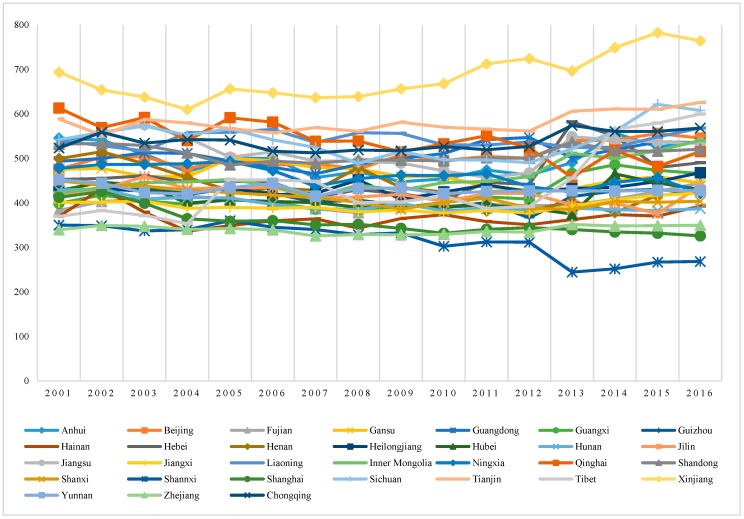
Per capita water footprint of food consumption in each province (m^3^/year).

By using the data of the urban population, rural population and water footprint of their food consumption, the per capita water footprint of food consumption in each province can be obtained, as shown in Figure 3. The per capita water footprint of food consumption in Xinjiang was the highest at 682.96 m^3^ per year while Shaanxi was the lowest, only 315.10 m^3^ per capita per year. The residents of Xinjiang and Shaanxi were 21.29 million and 37.24 million, respectively. The difference of per capita water footprint is heavily related to residents’ diet structure. Compared with other provinces, Xinjiang residents consume more beef and mutton which with higher water footprint. Based on the consumption of 12 kinds of food, we analysed per capita water footprint of residents’ food consumption, however, the results were smaller than the actual water consumption. If we take Beijing for example, the per capita water footprint of residents’ food consumption was 1023.05 m^3^ in 2005 if we took 14 kinds of food into consideration [69]. 

Compared with the average per capita water consumption over the years, the water footprint of residents’ food consumption in half of the provinces is greater than that of the per capita water consumption [75]. Per capita water consumption is the consumption of physical water resources, including domestic water, agricultural water, industrial water and ecological water. The water footprint of food consumption includes the consumption of virtual water and physical water, while the consumption of invisible water is often ignored by people, but it is huge. By attaching great importance to virtual water consumption, we can realize the real consumption of water resources and make more reasonable allocations and spatial replacement of water resources. From the point of per capita water resource, China’s per capita renewable inland fresh water resource is 2062 m^3^, only 35% of the world average [76]. Moreover, the per capita water resources in Tianjin, Shandong, Beijing and Ningxia are far lower than the national average and faced serious water shortages. However, the water footprint of food consumption per capita in these provinces is relatively high. For example, the per capita water resources in Beijing was 143.66 m^3^ over the years, far lower than the country average, while the water footprint of food consumption was 506.19 m^3^ per capita. Therefore, the mangement of water footprint of food consumption should be combined with the water resources situation in different places.

### 3.4. Water Footprint Structure of Residents’ Food Consumption 

The water footprint of residents’ food consumption includes the complete green water, blue water and gray water. According to the calculation, the contribution rate of green water footprint is the largest, reaching 69.36%. See Table 2 for the green water, blue water and gray water footprints of each type of food consumed.

Green water is the main source of agricultural production in China, far greater than the blue water and gray water footprint. Green water is “invisible water” absorbed by plants from the soil [40]. Due to its relatively low or negligible opportunity cost [77] and low negative environmental externalities [78], people believe that green water is of low importance. However, as an important part of crop water consumption, green water plays a crucial role in measuring the impact of agricultural production on water ecological environment [77]. Green water supports plant production in terrestrial ecosystems, including rain-fed agriculture [79]. Green water also has an important impact on global agricultural production and food security [78]. Therefore, China’s agricultural production should pay attention to green water management and soil and water conservation, and minimize non-productive green water evaporation losses.

Gray water indicates water pollution and reflects the technical level of agricultural production to some extent [80,81]. When nitrogen pollution is taken into account, the gray water footprint of China’s agricultural products accounts for more than 20% of the total water footprint [80,81,82]. China’s agricultural production has such a high gray water footprint, which indicates that the nitrogen fertilizer application rate and leaching rate are relatively high, causing serious non-point source pollution. According to the data released by the first national survey of pollution sources, agricultural sources discharged a total of 270.46 million tons of nitrogen, 0.28 million tons of phosphorus and 13.24 million tons of COD (Chemical Oxygen Demand) in 2007, accounting for 57%, 68% and 36% of the total emissions of similar pollutants, respectively, far higher than industrial and domestic sources [83].

Besides improving the level of agricultural production and reducing the use of fertilizers and pesticides, the gray water footprint is also a focus for reducing the water footprint of agricultural production. Agricultural production should control the use of chemical fertilizers and pesticides through management means. In addition, lower cost and lower pollution biological control technology should be promoted. Encouraging the development of environment-friendly agriculture, promoting clean production, reducing pollution and protecting the ecological environment are suggested. Moreover, this encourages the effective use of agricultural technologies and actively learns from new agricultural technologies abroad, such as among the Trans-Pacific Partnership agreement member countries [81], and promote domestic technological reform. As for gray water treatment, all types of gray water have good biodegradability, and some chemical processes can be used for removing the suspended solids, organic materials and surfactants in low-strength gray water [84].

### 3.5. Water Footprint of Consumption of Each Food 

**Figure 4:**
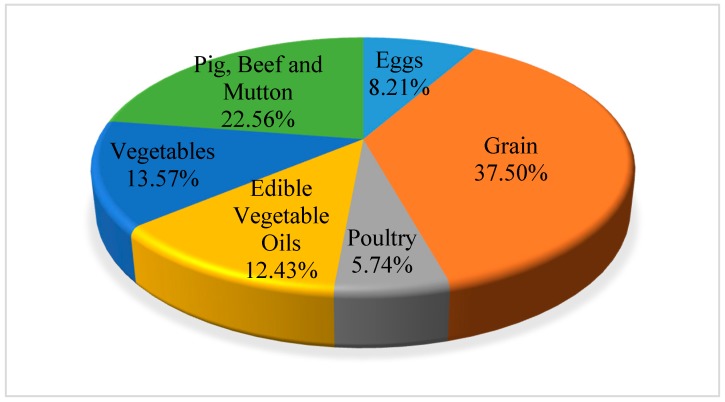
The proportion of water footprint consumed by each type of food in China.

**Figure 5:**
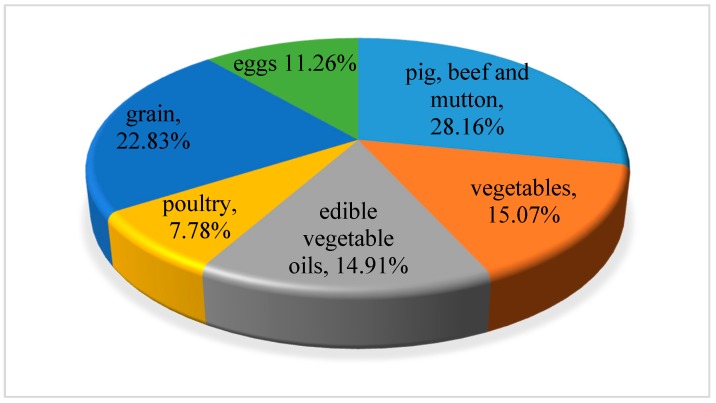
The proportion of water footprint consumed by each type of food of urban residents.

**Figure 6:**
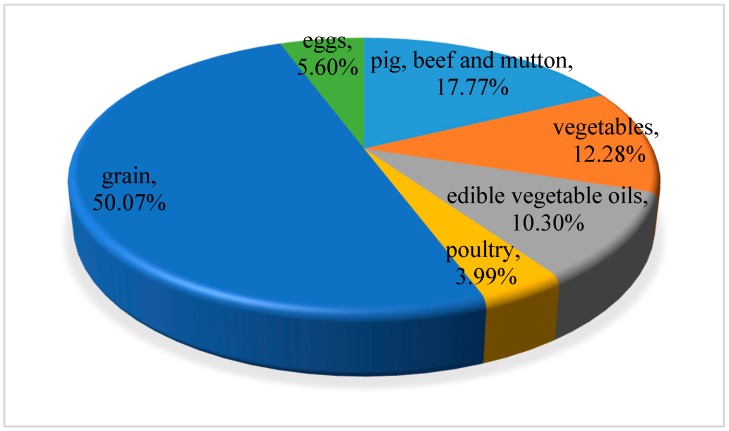
The proportion of water footprint consumed by each type of food of rural residents.

Since 2001, the water footprint of residents on food consumption of grain, vegetables, edible vegetable oil, meat, poultry and eggs has shown an increasing trend, and the increasing range of poultry, meat and eggs is large. Table 3 shows the water footprint of residents’ food consumption divided by food types over the years. From the perspective of food consumption structure, grain and pig, beef and mutton consumption contributed significantly to the water footprint of residents’ food consumption, contributing 37.5% and 22.56% to the total water footprint of food consumption. Vegetables and edible vegetable oils followed, and then followed by the water footprint of eggs and poultry. Based on Table 3, from 2001 to 2016, the proportion of water footprint consumed by each food in China of the total water footprint is shown in Figure 4. Figure 5 and Figure 6 showed the proportion of water footprint consumed by each type of food of urban and rural residents respectively. For urban residents, pig, beef and mutton consumption contributed the most to the total water footprint while grain consumption contributed the most among rural residents.

From the perspective of food-consumption structure, to reduce the overall food consumption water footprint of residents, the key is to reduce the consumption of grain and meat. Urban residents mainly pay attention to the reduction of meat consumption, while rural residents should pay more attention to the reduction of grain consumption. To reduce the water footprint of food consumption, it is necessary to uphold the concept of ecological consumption, balance the diet structure, and consciously control the consumption of food and meat and other water-intensive products. The concept of ecological consumption focuses on ecological needs based on the ecological environment, limits the consumption to the self-carrying capacity and purification capacity of the ecological environment, and aims at the coordinated development and harmonious coexistence between man and nature, which is the diversification of moderate consumption, green consumption and low carbon consumption. Ecological consumption not only requires ecological consumption motivation, but also ecological consumption processes and results [85,86]. Residents are direct participants in consumption. They should improve their awareness of ecological and environmental responsibility, actively change their consumption concept, and advocate green and low-carbon consumption.

On the other hand, a big problem related to food consumption is food waste. A large amount of food is lost or wasted in the process of production and consumption [87]. In 2010, for example, China lost or wasted about 19% of its grain, equivalent to wasting 135 billion m^3^ of water footprint. Consumer waste is the largest part of total food waste [88]. Therefore, to reduce the water footprint, we need to pay attention to reducing food waste. If residents are willing to reduce food waste by an average of one tablespoon (about 5 grams) per day, China will add 2.6 million tons of grain and save 1.79 billion m^3^ of water footprint every year [87].

## 4. Conclusions

This paper aims to undertake a comprehensive analysis on the water footprint of food consumption in China from the perspectives of urban and rural residents, per capita water footprint, water footprint structure and food consumption structure. We undertook a detailed analysis on each province. This is helpful for improving the water management and food consumption structure of each province. 

Overall, from 2001 to 2016, the total water footprint of residents’ food consumption was 9681.86 billion m^3^ and the average was 605.12 billion m^3^/year, basically showing an upward trend. Guangdong residents have the highest water footprint for food consumption, at 49.37 billion m^3^/year due to the highest population and higher consumption of water-intensive foodstuffs such as grain and meat in their diet, followed by Shandong and Sichuan. The water footprint of Xizang residents’ food consumption was the lowest, at only 1.70 billion m^3^/year, due to it having the least population, followed by Ningxia and Qinghai. From the rural and urban perspective, the water footprint of food consumption consumed by urban residents was on the rise while that consumed by rural residents was on the decline in China, which was consistent with the changing trend of the population. The rural population consumed more virtual water embedded in food than the urban population. The per capita water footprint of food consumption in Xinjiang was the highest at 682.96 m^3^ per year while Shaanxi was the lowest at only 315.10 m^3^ per capita per year. Compared with the average per capita water consumption over the years, the water footprint of residents’ food consumption in half of the provinces is greater than that of the per capita water consumption. From the water footprint structure level, the contribution rate of the green water footprint was the largest, reaching 69.36%. The second was the gray water footprint, accounting for 18.71%, and the blue water footprint accounting for 11.93%. From the perspective of food consumption structure, grain and pig, beef and mutton consumption contributed significantly to the water footprint of residents’ food consumption, contributing 37.5% and 22.56% respectively.

## Figures and Tables

**Table 1:** Data explanation of water footprint per unit of food consumed.

Agricultural Categories	Products	Water Footprint
Crop products	Rice	Directly from report No. 47
	Wheat	Directly from report No. 47
	Corn	Directly from report No. 47
	Soybean	Directly from report No. 47
	Potato	Directly from report No. 47
	Vegetables	Average of 20 kinds of vegetables (cabbage, artichokes, asparagus, lettuce and chicory, spinach, tomato, cauliflower and broccoli, squash and bottle gourd, cucumber, eggplant, onion and onion, dried onion, garlic, green beans, peas, beans, carrots and white radish, corn, and other fresh vegetables)
	Edible vegetable oil	Average of 11 kinds of vegetable oil (soybean oil, peanut oil, palm oil, olive oil, castor oil, sunflower oil, rapeseed oil, sesame oil, cottonseed oil, linseed oil, corn oil)
Animal products	Pork, beef, mutton, poultry and eggs	According to the ratio with the national average which was from report No. 48

**Table 2:** Water footprint structure of the food consumed (billion m^3^).

Water Footprint Structure	Eggs	Grain	Poultry	Edible Vegetable Oil	Vegetables	Pig, Beef and Mutton	Total	Proportion
Green water	566.28	2062.67	396.52	999.08	822.23	1868.64	6715.42	69.36%
Blue water	57.82	846.23	39.35	47.08	35.77	129.12	1155.38	11.93%
Gray water	170.56	721.78	119.42	156.89	455.66	186.76	1811.06	18.71%

**Table 3:** Water footprint of residents’ food consumption divided by food types (million m^3^).

Year	Eggs	Grain	Poultry	Edible Vegetable Oil	Vegetables	Pig, Beef and Mutton
2001	41.5	276.06	23.21	60.71	83.75	105.48
2002	42.51	271.64	26.71	63.64	85.3	113.97
2003	44.95	260.68	28.03	60.84	85.41	121.24
2004	42.94	254.36	26.38	58.39	85.89	120.89
2005	45.13	237.27	31.59	63.32	82.68	131.16
2006	46.88	232.36	30.23	64.01	82.42	133.29
2007	46	223.31	31.22	66.91	82.06	124.71
2008	50.19	221.06	34.06	71.97	85.6	124.25
2009	50.45	213.42	34.8	71.08	84.02	134.71
2010	49.2	208.63	36.07	70.07	82.35	140.12
2011	52.12	201.38	39.95	77.97	81.35	144.44
2012	55.08	194.71	40.71	79.68	80.01	146.83
2013	52.31	212.67	37.31	95.6	76.45	153.22
2014	54.17	207.56	41.66	104.34	78.18	157.85
2015	59.42	209.32	44.63	97.8	77.71	164.41
2016	61.8	206.26	48.73	96.74	80.49	167.94
